# Draft Genome Sequences of Three Sponge-Associated Actinomycetes Exhibiting Antimycobacterial Activity

**DOI:** 10.1128/MRA.00858-19

**Published:** 2019-08-22

**Authors:** Daniela Tizabi, Ana Sosa, Tsvetan Bachvaroff, Russell T. Hill

**Affiliations:** a Institute of Marine and Environmental Technology, University of Maryland Center for Environmental Science, Baltimore, Maryland, USA; Georgia Institute of Technology

## Abstract

Marine actinomycetes (order *Actinomycetales*) are of interest as a promising source of pharmaceuticals. The genomes of three novel sponge-associated actinomycetes exhibiting antimycobacterial activity, Brevibacterium sp. strain XM4083, Micrococcus sp. strain R8502A1, and Micromonospora sp. strain XM-20-01, were sequenced in an effort to identify compounds responsible for growth-inhibiting activity.

## ANNOUNCEMENT

Actinomycetes produce an extensive arsenal of compounds with pharmaceutical potential (1–4). Approximately 70% of all known antibiotics are derived from actinomycetes (5). These bacteria exist in a diverse range of environments, both terrestrial and aquatic (3–5). Marine actinomycetes are of particular interest to drug discovery efforts, as advances in culturing techniques increasingly facilitate access to previously underexplored rare species (3, 4). In the case of sponge symbionts, the compounds they produce may aid in the defense of their nonmotile hosts. Novel strains of Brevibacterium, Micrococcus, and Micromonospora isolated from the giant barrel sponge Xestospongia muta showed promising growth inhibition against several Mycobacterium spp. in a screen performed as part of this study. Representatives from these genera produce secondary metabolites with potent bioactivity (1, 6–12). Genomic analysis was performed to elucidate the bioactive capabilities of these bacteria and predict which bioactive compound(s) may be produced by these strains.

Microbial strains were previously isolated from X. muta samples collected by SCUBA divers in Conch Reef, Key Largo, FL, in July 2001 and June 2004 (13) and stored at −80°C. Strains were assigned taxonomic classifications after initial isolation, and these were confirmed at the time of this study on the basis of 16S rRNA gene sequence analysis. Strains were cultured in International *Streptomyces* Project 2 (ISP2) medium for 10 to 16 days and incubated at 30°C with shaking at 150 rpm. Organic extracts were prepared by ethyl acetate extraction and produced zones of inhibition in a disk diffusion assay against at least one of three test strains, Mycobacterium smegmatis MC^2^ 155, Mycobacterium marinum 927, and Mycobacterium tuberculosis H37Ra. DNA was extracted from the *Brevibacterium* and *Micromonospora* strains using the UltraClean microbial DNA isolation kit. Phenol-chloroform extraction was required for *Micrococcus* DNA isolation. Genomic DNA was sequenced on the MiSeq sequencer (Illumina) using the MiSeq version 2.4.0.4 reagent kit (300 cycles for *Brevibacterium* sp. and *Micromonospora* sp. and 500 cycles for *Micrococcus* sp.). The Nextera DNA Flex library prep kit (100 ng DNA) was used to prepare the libraries for the *Brevibacterium* and *Micromonospora* strains, while the Nextera XT library prep kit (1 ng DNA) was used to prepare the *Micrococcus* library. Reads were assembled *de novo* using SPAdes version 3.11.1 (14), which includes a built-in BayesHammer read error correction tool. Contigs were filtered primarily based on coverage, followed by match identity after comparison with the Nucleotide BLAST database. Genome annotation was performed using PATRIC version 3.5.41 (15), and biosynthetic gene clusters (BGCs) were identified using antiSMASH version 5.0 (16) (Table 1). Default parameters were used for all software programs.

**TABLE 1:** Biosynthetic gene clusters identified for novel actinomycetes with antiSMASH

Strain	Pigment	No. of BGCs identified^*a*^	Order of BGCs (similarity to known cluster [%])
*Brevibacterium* sp. XM4083	Yellow	2	Carotenoid (57), ectoine (75)
*Micrococcus* sp. R8502A1	Yellow	1	Carotenoid (66)
*Micromonospora* sp. XM-20-01	Initially pink, then black	7	Diazepinomicin (75), sioxanthin (100), alkyl-*O*-dihydrogeranyl-methoxyhydroquinones (71), sioxanthin (100), micromonolactam (100), micromonolactam (100), micromonolactam (100)

a Only BGCs with at least 40% similarity to known clusters on antiSMASH were considered.

For *Brevibacterium* sp. strain XM4083, MiSeq sequencing generated 4,324,102 read pairs, from which SPAdes assembled 102 contigs. Based on coverage, the final assembly contained 13 contigs ranging in size from 5,527 bp to 1,292,640 bp (*N*_50_, 761,026 bp), with average k-mer coverage of 192-fold, yielding a genome of ∼4.0 Mb with a G+C content of 68.02%. Annotation with PATRIC identified 3,782 genes, including 3,732 protein-coding sequences (CDS), 47 tRNA genes, and 3 rRNA genes.

For *Micrococcus* sp. strain R8502A1, MiSeq sequencing generated 2,607,861 read pairs, from which SPAdes generated 609 contigs. The final assembly contained 128 contigs ranging in size from 239 bp to 137,025 bp (*N*_50_, 38,085 bp), with average k-mer coverage of 158-fold, yielding a genome of ∼2.4 Mb with a G+C content of 72.91%. Annotation with PATRIC identified 2,373 genes, including 2,322 CDS, 49 tRNA genes, and 2 rRNA genes.

For *Micromonospora* sp. strain XM-20-01, MiSeq sequencing generated 1,565,813 read pairs, from which SPAdes generated 30,851 contigs. The final assembly contained 149 contigs ranging in size from 250 bp to 343,851 bp (*N*_50_, 105,009 bp), with average k-mer coverage of 32-fold, yielding a genome of ∼6.7 Mb with a G+C content of 72.86%. Annotation with PATRIC identified 6,401 genes, including 6,347 CDS, 51 tRNA genes, and 3 rRNA genes.

### Data availability.

This whole-genome shotgun project was deposited at DDBJ/ENA/GenBank under the accession no. VFYQ00000000, VIGS00000000, and VFYR00000000 for *Micromonospora* sp. XM-20-01, *Micrococcus* sp. R8502A1, and *Brevibacterium* sp. XM4083, respectively. Raw reads for *Micromonospora* sp. XM-20-01, *Micrococcus* sp. R8502A1, and *Brevibacterium* sp. XM4083 were deposited in the NCBI SRA under the accession no. SRR9208918, SRR9208919, and SRR9208920, respectively. They pertain to BioProject accession no. PRJNA543824.
